# Essential oil composition and antinociceptive activity of *Thymus capitatus*

**DOI:** 10.1080/13880209.2017.1279672

**Published:** 2017-01-19

**Authors:** Juan Carlos Ramos Gonçalves, Danilo Andrade de Meneses, Aliny Pereira de Vasconcelos, Celyane Alves Piauilino, Fernanda Regina de Castro Almeida, Edoardo Marco Napoli, Giuseppe Ruberto, Demetrius Antônio Machado de Araújo

**Affiliations:** a Núcleo de Pesquisas em Plantas Medicinais, Universidade Federal do Piauí, Teresina, Brazil;; b Centro de Biotecnologia, Universidade Federal da Paraíba, João Pessoa, Brazil;; c Consiglio Nazionale delle Ricerche, Istituto di Chimica Biomolecolare, Catania, Italy

**Keywords:** Pain, glutamate, action potential

## Abstract

**Context:** The essential oil (EO) from *Thymus capitatus* Hoff. et Link. (Lamiaceae) has been traditionally used for its medicinal properties, such as anti-inflammatory, analgesic, antioxidant and antimicrobial properties.

**Objective:** Characterize the constituents from *T. capitatus* EO and further evaluate the antinociceptive activity by *in vivo* and *in vitro* procedures.

**Materials and methods:** Gas chromatography–mass spectrometry was used to identify and quantify the constituents of the *T. capitatus* EO. The antinociceptive activity was evaluated *in vivo* by the glutamate-induced nociception model in male Swiss mice (25 g), at doses of 3, 6 and 12 mg/kg, 1 h before evaluation of the licking time response (0–15 min). The mechanism of *T. capitatus* EO (1–500 μg/mL) on the isolated nerve excitability of Wistar rat (300 g) was assessed by the single sucrose technique.

**Results and discussion:** The EO of *T. capitatus* presented 33 components, mainly monoterpenes and sesquiterpenes, carvacrol (*ca.* 80%) was its major constituent. *T. capitatus* EO induced antinociception in orally treated mice (3, 6, and 12 mg/kg) reducing the licking time from control (100.3 ± 11.9 s) to 84.8 ± 12.2, 62.7.6 ± 9.9, and 41.5 ± 12.7 s, respectively (*n* = 8; *p* < 0.05). Additionally, we have demonstrated that *T. capitatus* EO (500 μg/mL) decreased the compound action potential amplitude (*V_CAP_*) of about 80.0 ± 4.3% from control recordings (*n* = 4; *p* < 0.05). Such activity was presumably mediated through a voltage-gated Na^+ ^channels.

**Conclusions:** The present study demonstrated the antinociceptive activity of *Thymus capitatus* essential oil, which acts via peripheral nervous excitability blockade.

## Introduction

The essential oils (EO) are volatile molecules generated by the secondary metabolism of higher plants and are produced by specialized secretory structures, such as glandular hairs and differentiated parenchyma cells (Lane et al. 2010). EO constituents belong mainly to two chemical groups: terpenoids (monoterpenes and sesquiterpenes of low molecular weight) and, to a lesser extent, phenylpropanoids (Regnault-Roger et al. 2012). These EO also have shown many biological activities, such as analgesic, anticonvulsant, anxiolytic, antioxidant, and antimicrobial properties (Faturi et al. 2010; Pergolizzi et al. 2010; Almeida et al. 2011).

*Thymus* (Lamiaceae) comprises over 300 species of hardy perennial herbaceous plants and subshrubs, which are native to southern Europe, Asia and North Africa (Tepe et al. 2004). Thyme is a distinguished condiment that is used in traditional medicine for several purposes, for example, as an anti-inflammatory and analgesic agent (Alamger et al. 2015; Amirghofran et al. 2016). The pharmacological activity of *Thymus capitatus* Hoff. et Link. EO have been demonstrated, including antiseptic, antioxidant, and antimicrobial properties (Consentino et al. 1999; Al-Mustafa & Al-Thunibat 2008; Mkaddem et al. 2010; Russo et al. 2013; Džamić et al. 2015), however, the antinociceptive and analgesic properties have not yet been evaluated. Therefore, the present study was designed to determine the major constituents from the *T. capitatus* EO and to evaluate its antinociceptive effects by *in vivo* and *in vitro* procedures.

## Materials and methods

### Chemicals

Pure standards for GC-MS analysis were purchased from Sigma-Aldrich (St. Louis, MO) and Extrasynthese (Lyon, France). Glutamate, morphine, Tween 80 (polyoxyethylene sorbitan monooleate), and dimethyl sulfoxide (DMSO) were purchased from Sigma-Aldrich (St. Louis, MO). All salts used in physiological solution, sucrose, glucose and NaOH, were purchased from Vetec (Rio de Janeiro, Brazil). HEPES [4-(2-hydroxyethyl)-1-piperazineethanesulfonic acid] was acquired from Amresco (Solon, OH).

### Plant processing

Wild Thyme sample was collected in the middle of Sicily, in 2006, at bloom stage and dried at room temperature until no constant weight loss was observed at the collection point, Calascibetta lands (Enna province, Italy), 37°59′83″(N), 14°25′26″(E). The plant collection and identification was made by the Regional Unit UOT Enna, as described by Napoli et al. (2010), in which herbarium voucher specimen was deposited. Dried aerial part of the sample (100 g) was subjected to hydrodistillation in accordance with current European Pharmacopoeia 6.0 (2008) until there was no significant increase in the volume of oil collected (3 h). The essential oil obtained was dried over anhydrous sodium sulfate and stored under N_2_ in a sealed vial until required.

### Oil analysis

Gas chromatographic (GC) analyses were run on a Shimadzu gas chromatograph, Model 17-A, equipped with a flame ionization detector (FID) and with an operating software Class VP Chromatography Data System, version 4.3 (Shimadzu Corporation, Kyoto, Japan). Analytical conditions were SPB-5 capillary column (15 m × 0.10 mm × 0.15 μm) and helium as the carrier gas (1 mL/min). Injection was done in split mode (1:200), injected volume was 1 μL (4% essential oil/CH_2_Cl_2_ v/v), and the injector and detector temperatures were 250 °C and 280 °C, respectively. Linear velocity in column 19 was cm/s. The oven temperature was held at 60 °C for 1 min, then programed from 60 to 280 °C at 10 °C/min then 280 °C for 1 min. Percentages of compounds were determined from their peak areas in the GC-FID profiles.

Gas chromatography-mass spectrometry (GC-MS) analysis was carried out in the fast mode on a Shimadzu GC-MS model GCMS-QP5050A, with the same column and the same operative conditions used for the analytical GC and the operating software GCMS solution version 1.02 (Shimadzu Corporation, Kyoto, Japan). We adjusted the ionization voltage at 70 eV, the electron multiplier at 900 V and the ion source temperature at 180 °C. Mass spectra data were acquired in the scan mode in an *m/z* range of 40–400. The same oil solutions (1 μL) were injected with the split mode (1:96).

The compounds were identified based on their GC retention index (relative to C9-C22 *n*-alkanes on the SPB-5 column), computer matching of spectral MS data with those from NIST MS 107 and NIST 21 libraries (National Institute of Standard and Technology 1998), comparison of the fragmentation patterns with those reported in the literature (Adams 2001) and, whenever possible, co-injections with authentic samples.

### Animals

Adult male Swiss mice and Wistar rats, weighing around 25 and 300 g, respectively, were randomly housed in appropriate cages at 24 ± 2 °C on a 12 h light cycle with free access to food (Purina, São Paulo, Brazil). All procedures in this study were carried out in accordance with Institutional Animal Care and Use Committee (CEEA-UFPI and CEPA-UFPB, Brazil).

### Glutamate-induced nociception

The procedure used was similar to that previously described by Beirith et al. (2002). In brief, a volume of 20 μL of glutamate (10 μM/paw) was injected intraplantarly in the ventral surface of the mice’s right hind paw. Animals were divided into five groups (*n* = 8), orally treated with vehicle (Tween 80 0.1% in distilled water) in the control group, *T. capitatus* EO (3, 6, and 12 mg/kg) and morphine (5 mg/kg), 1 h before experimentation. After drug treatment, the licking time (0–15 min) of the glutamate-injected paw was recorded and considered as indicative of nociception (Donato et al. 2015).

### Electrophysiological experiments

The single sucrose gap technique was used as previously described by Gonçalves et al. (2010). Briefly, rat sciatic nerves (*n* = 4) were carefully removed and immediately positioned in an experimental chamber composed of five compartments (*I–V*), electrically isolated by solid vaseline. All compartments were filled with a physiological solution, composed of (in mM): NaCl 150; KCl 4; CaCl_2_ 2; MgCl_2_ 1; glucose 10 and HEPES 10. The pH was adjusted to 7.4 with NaOH. Compartments *I* and *II* of the chamber were used to apply supramaximal stimulation (6–10 V/100 ms), manually triggered at 5 min intervals (CF Palmer, London, UK). The compartment *III* was superfused by isotonic sucrose (290 mM; 1 mL/min) to electrically isolate the neighbouring recording compartments. Compartments *IV* and *V* were used for drug perfusion (1 mL/min) and data recording, acquired by a microcomputer-based 12-bit A/D converter (10.5 kHz) and later analyzed by proper software (Lynx, São Paulo, Brazil)).

*T. capitatus* EO was diluted into a vehicle (0.1% Tween 80 in physiological solution) at concentrations of 1, 100 and 500 μg/mL. Drug incubation was performed for 30 min followed by nerve washout with physiological solution for additional 30 min. To quantify drug effects, we analyzed the compound action potential (CAP) amplitude (*V_CAP_*, in mV) by measuring the difference between the baseline and the maximal voltage achieved. The CAP depolarization velocity (*DV_CAP_*, in V/s) was the rate between *V_CAP_* and the time required to reach the CAP peak. The time constant of repolarization (*τ_rep_*, in ms) was calculated by the equation: *V = V*0*exp(*−t/τ*), as described by Alves et al. (2010).

### Statistical analysis

Data are presented as the mean ± S.E.M. of independent experiments. To assess the significance level, analysis of variance (ANOVA) followed by Dunnet’s test for *in vivo* data (*n* = 8) and the two-tailed Student’s *t*-test for *in vitro* data (*n* = 4) were used. Differences between experimental and control groups were considered significant when *p <* 0.05.

## Results

Table 1 lists the composition of the *T. capitatus* essential oil. In total, 33 components were fully identified, covering more than 96% of the total composition. Monoterpenes, both hydrocarbons and oxygenated, were the most highly represented classes, while sesquiterpenes and other classes were the least represented.

**Table 1.** Chemical composition of *T. capitatus* essential oil (EO).

			*T. capitatus* EO
			#	KI^a^	Compound	Area^b^ (%)
1	778	2-Methyl-butanoic acid methyl ester	0.1
2	932	α-Thujene	0.7
3	939	α-Pinene	0.6
4	954	Camphene	0.2
5	982	β-Pinene	0.4
6	993	β-Myrcene	1.7
7	1008	α-Phellandrene	0.2
8	1015	*p*-Menth-1-(7),8-diene	0.1
9	1021	α-Terpinene	1.2
10	1033	*p*-Cymene	4.4
11	1035	Limonene	0.4
12	1052	*trans*-β-Ocimene	0.1
13	1063	γ-Terpinene	3.7
14	1072	Sabinene Hydrate	0.2
15	1091	α-Terpinolene	0.2
16	1100	Linalool	0.9
17	1126	α-Campholenal	0.1
18	1172	Borneol	0.4
19	1181	Terpinen-4-ol	0.8
20	1193	α-Terpineol	0.1
21	1248	Nerol	0.1
22	1255	Neral	0.1
23	1264	Carvone	0.1
24	1273	Geranial	0.1
25	1290	Thymol	0.3
26	1313	Carvacrol	79.9
27	1362	Thymol acetate	0.1
28	1425	Caryophyllene	2.3
29	1446	Aromadendrene	0.1
30	1460	α-Humulene	0.1
31	1511	β-Bisabol	0.2
32	1545	α-Cadinene	0.2
33	1589	Caryophyllene Oxide	0.2
Monoterpene hydrocarbons			13.7
Oxygenated monoterpenes			83.0
Sesquiterpenes			3.1
Others			0.1

KI: Kovat’s indices; RT: retention time.

a Retention index (KI) relative to standard mixture of *n*-alkanes on SPB-5 column.

b The numbering refers to elution order, and values (relative peak area percent) represent averages of 3 determinations.

We stated that the oxygenated monoterpene carvacrol (*ca.* 80%) strongly characterizes the *T. capitatus* EO being its major constituent. The two monoterpene hydrocarbons, namely *p*-cymene (*ca.* 4.4%) and γ-terpinene (*ca.* 3.7%) biosynthetically correlated to carvacrol, were the other two main components, although in lower concentrations (Table 1). Finally, among the other components, only the sesquiterpene caryophyllene reaches an appreciable amount (*ca.* 2.3%).

**Figure 1.**
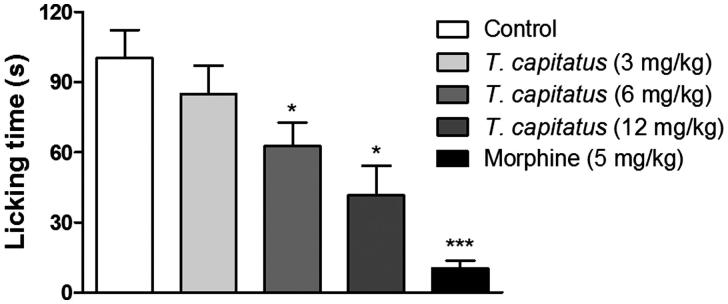
Antinociceptive effect of *T. capitatus* EO in mice. The *T. capitatus* EO was administered in mice orally (3–12 mg/kg) 1 h before initiating the glutamate-induced nociception method. Control animals were treated with vehicle alone. Morphine (5 mg/kg) was used as a positive control. Values are expressed as mean ± S.E.M, *n* = 8. *p < 0.05, ***p < 0.001 vs. control (ANOVA followed by Dunnet’s test).

During the test of glutamate nociception, we observe that animals orally treated with *T. capitatus* EO at doses of 3, 6, and 12 mg/kg (Figure 1), reduced the licking time from control (100.3 ± 11.9 s) to 84.8 ± 12.2, 62.7.6 ± 9.9 and 41.5 ± 12.7 s (*p* < 0.05), respectively. As expected, the standard drug morphine (5 mg/kg) decreased the nociceptive response to 10.3 ± 3.4 s from control (*p* < 0.05).

**Figure 2.**
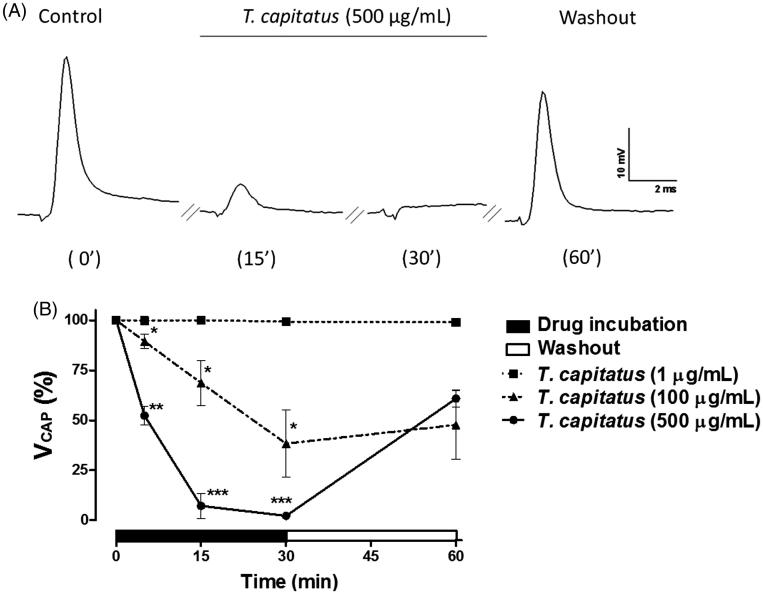
*T. capitatus* EO reversibly blocks the peripheral nerve excitability. (A) Representative CAP recordings obtained by the single sucrose gap technique after 5, 15 and 30 min of *T. capitatus* EO (500 μg/mL) incubation. After drug incubation, the nerve was washed out with the physiological solution for 30 min. Control records were obtained when sciatic nerves were submitted to the physiological solution only. Stimulation parameters were 6–10 V/0.1 ms. (B) Time- and concentration-dependent effects of *T. capitatus* EO (1–500 μg/mL) on CAP amplitude (*V_CAP_*) during 30 min incubation followed by nerve washout (30 min). Values are expressed as mean ± S.E.M, *n* = 4. *p < 0.05; **p < 0.01; ***p < 0.001 vs. control (Student’s *t*-test).

Testing the *T. capitatus* EO in the peripheral nerve excitability, we showed that 500 μg/mL was sufficient to completely block *V_CAP_* after 30 min of incubation and this effect was latter reverted by 30 min of drug removal (Figure 2(A)). In fact, 500 μg/mL of *T. capitatus* EO practically reached is maximum effect with only 15 min of incubation (Figure 2(B)). At 100 μg/mL of *T. capitatus* EO, we observed that *V_CAP_* was reduced to 10.6 ± 3.6, 31.4 ± 11.3, and 58.1 ± 16.8% from control, after 5, 15, and 30 min of incubation, respectively (Figure 2(B)). Vehicle alone did not induce any significant change in the CAP characteristics (data not shown).

**Figure 3.**
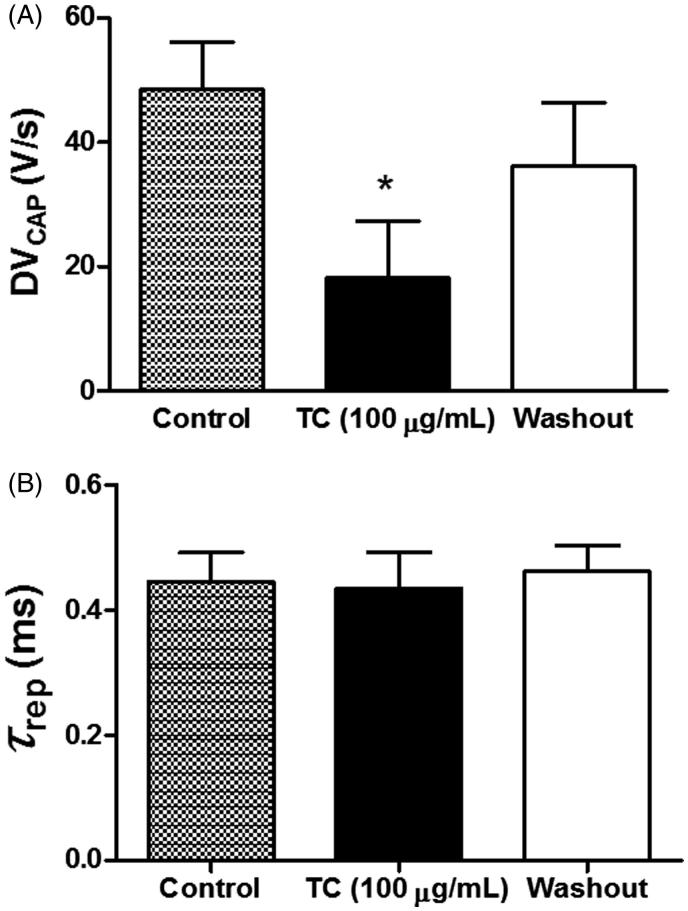
Effects of *T. capitatus* on the depolarization and repolarization phases of the CAP. *T. capitatus* EO (TC, 100 μg/mL) was incubated in the rat sciatic nerve for 30 min followed by drug washout out with the physiological solution. The CAP parameters like (A) the depolarization velocity (*DV_PAC_*), and (B) the time constant of repolarization (*τ_rep_*), were quantified and compared to control. Values are expressed as mean ± S.E.M, *n* = 4. *p < 0.05 vs. control (Student’s *t*-test).

We also evaluated the effect of *T. capitatus* EO on *DV_CAP_* and *τ_rep_* parameters in order to assess for alterations in the depolarization and/or repolarization phases of CAP recordings. Taken together, our results showed that *T. capitatus* EO (100 μg/mL) decreased *DV_CAP_* from 48.4 ± 7.6 V/s (control) to 18.1 ± 9.1 V/s (*p* < 0.05) after 30 min of incubation. Such effect was reversed to 26.1 ± 10.2 V/s after drug washout (Figure 3(A)). Meanwhile, no differences were observed on the CAP repolarization phase, measured by *τ_rep_* (Figure 3(B)).

## Discussion

The aforementioned aromatic profile is similar to Sicilian Thyme, as recently reported in a study on 30 samples collected throughout the Sicilian island, suggesting that *Thymus capitatus* [syn. *Coridothymus capitatus* (L.) Rchb.f., *Satureja capitata* L., *Thymbra capitata* (L.) Cav.] is the most widespread wild species in the Sicilian area (Napoli et al. 2010). In the present study, we analyzed the EO composition of *T. capitatus*, demonstrating that it is composed of 33 different molecules, including monoterpenes and sesquiterpenes, and other compounds like phenols, alcohols, organic acids, aldehydes and ketones. The monoterpene carvacrol was found as the main component, corresponding to ∼80% of the whole oil (Table 1).

Recently, several studies have reported the antinociceptive activity of *Thymus* sp. extracts and their major constituents (Mahmoudi et al. 2011; Taherian et al. 2009; Cavalcante Melo et al. 2012). In this way, we evaluated whether *T. capitatus* EO also induces analgesy *in vivo*. For that, the glutamate-induced nociception model was performed in mice pretreated with *T. capitatus* EO by the oral tract, in order to imply that the drug was absorbed *in vivo*. Our data demonstrated significant analgesic activity and dose-dependency of *T. capitatus* EO (Figure 1).

At the peripheral level, the glutamate is involved in nociceptive transmission through primary afferent fibres, as well as in the development and maintenance of the pain response (Osikowicz et al. 2013; Bardoni 2013). This amino acid acts on ionotropic receptors such as *N*-methyl-d-aspartate (NMDA), increasing cation influx, like Na^+ ^or Ca^2+^, through plasma membrane, activating post-synaptic transmission and deflagrating action potentials in nociceptive fibres, leading to pain sensation (Beirith et al. 2002; Srebro et al. 2015). Therefore, it is plausible to suggest that the constituents of *T. capitatus* EO might also interfere with the excitability of peripheral nerves.

To assess the effects of *T. capitatus* EO on the isolated nerve excitability, we performed the single sucrose technique. This method enables the study of bioactive compounds on the peripheral nerve by measuring the CAP evoked by external electrodes (Alves et al. 2010; Gonçalves et al. 2010). According to the presented data, we have demonstrated that *T. capitatus* EO impede nervous transmission by blocking the CAP amplitude, in a concentration- and time-dependent manner (Figure 2(A) and (B)). In addition, such effect was reverted by washing the drug out. These data also suggest that *T. capitatus* EO did not damage the nerve fibres functionality during the experimentation period.

The CAP recording is considered the algebraic sum of the action potential from all individual fibres of the nerve. Their shapes and properties indicate a single-action potential and therefore it can be a useful tool for an initial searching of neuroactive drug candidates. It has also been shown that inhibition of neuronal excitability, observed as a decrease in the CAP amplitude, might be associated with the blockade of the voltage-gated Na^+ ^channels (Na_v_), which are responsible for the rising phase of the action potential in neurons (Araújo et al. 2011). The present study demonstrated that 100 μg/mL of *T. capitatus* EO delayed the CAP depolarization phase (*DV_CAP_*) after 30 min-incubation in rat sciatic nerves. Thus, we can suggest a Na_v_ blocking-like effect promoted by *T. capitatus* EO in a reversible manner, since this effect was recovered by drug washout (Figure 3(A)). In fact, recent findings have demonstrated the ability of carvacrol, the major constituent of *T. capitatus* EO, to induce Na_v_ blockade in DRG neurons (Joca et al. 2012, 2015). Those findings corroborate our results and strongly suggest that carvacrol is the main active molecule behind the antinociceptive effects of *T. capitatus*.

On the other hand, the voltage-gated K^+ ^channels are believed to be an important factor in the repolarization phase of peripheral nerves, especially the delayed rectifier potassium channel (K_v_). Some studies have demonstrated that the time constant of depolarization (*τ_rep_*) of the CAP can be used as a valuable tool for the investigation of potential K_v_ blockers (Alves et al. 2010). Hence, it is unlikely that *T. capitatus* EO is related to this channel variety since no change in *τ_rep_* was observed (Figure 3(B)).

In conclusion, our study reported that the carvacrol-rich essential oil from *T. capitatus* exerts its antinoceptive activity through peripheral nervous excitability blockade.
